# Theiler's Murine Encephalomyelitis Virus as a Vaccine Candidate for Immunotherapy

**DOI:** 10.1371/journal.pone.0020217

**Published:** 2011-05-20

**Authors:** Kevin D. Pavelko, Megan A. Girtman, Yoshihiro Mitsunaga, Yanice V. Mendez-Fernandez, Michael P. Bell, Michael J. Hansen, Kathleen S. Allen, Moses Rodriguez, Larry R. Pease

**Affiliations:** 1 Department of Immunology, Mayo Clinic, Rochester, Minnesota, United States of America; 2 Department of Neurology, Mayo Clinic, Rochester, Minnesota, United States of America; University Paris Sud, France

## Abstract

The induction of sterilizing T-cell responses to tumors is a major goal in the development of T-cell vaccines for treating cancer. Although specific components of anti-viral CD8+ immunity are well characterized, we still lack the ability to mimic viral CD8+ T-cell responses in therapeutic settings for treating cancers. Infection with the picornavirus Theiler's murine encephalomyelitis virus (TMEV) induces a strong sterilizing CD8+ T-cell response. In the absence of sterilizing immunity, the virus causes a persistent infection. We capitalized on the ability of TMEV to induce strong cellular immunity even under conditions of immune deficiency by modifying the virus to evaluate its potential as a T-cell vaccine. The introduction of defined CD8+ T-cell epitopes into the leader sequence of the TMEV genome generates an attenuated vaccine strain that can efficiently drive CD8+ T-cell responses to the targeted antigen. This virus activates T-cells in a manner that is capable of inducing targeted tissue damage and glucose dysregulation in an adoptive T-cell transfer model of diabetes mellitus. As a therapeutic vaccine for the treatment of established melanoma, epitope-modified TMEV can induce strong cytotoxic T-cell responses and promote infiltration of the T-cells into established tumors, ultimately leading to a delay in tumor growth and improved survival of vaccinated animals. We propose that epitope-modified TMEV is an excellent candidate for further development as a human T-cell vaccine for use in immunotherapy.

## Introduction

Theiler's murine encephalomyelitis virus (TMEV) is a natural mouse pathogen that has been used to identify the T-cell effector molecules necessary for clearance of central nervous system infection. These findings show that viral clearance depends on CD8+ α/β T-cell interactions with viral peptides presented in the context of specific MHC class I alleles [1], [2], with the best example being the response to VP2_121–130_ in the context of H-2D^b^ [3], [4]. Further, these strong T-cell responses can be elicited in the absence of CD4+ T-cells, CD28 co-stimulation and interferon gamma [5]. The ability to drive strong CD8 T-cell immunity in a variety of immune deficient states makes TMEV an attractive vaccine vector for eliciting CD8 T-cell responses to tumor antigens. Previous work has shown that the introduction of fluorescent proteins into the genome of TMEV can be used as a tool to track cells infected with this virus and disruption of the viral genome does not block its ability to infect cells [6]. Further, the introduction of MHC class II restricted self antigens into the virus has been used as a way to enhance the autoimmune attack incurred through infection of the central nervous system with recombinant TMEV [7]. The current work examines the ability of a modified TMEV vaccine to direct robust systemic CD8-restricted anti-tumor immunity to introduced antigens.

The ideal viral vaccine has not yet been established. Several viruses have been adapted as potential carriers of antigen for eliciting targeted anti-tumor immunity [8], [9]. However, these viruses are often composed of large genomes whose constituents are often used to avoid immune detection [10], [11]. We have introduced the MHC class I model peptide OVA_257_ into the coding sequence of the 8093 base pair genome of TMEV (TMEV-L/OVA) for use as a potential anti-tumor vaccine. Despite some attenuation due to genetic modification, this relatively small virus can efficiently generate CD8+ cytotoxic T-cell responses to the introduced antigen and inhibit tumor growth through the specific induction of tumor infiltrating lymphocytes. This new virus vaccine provides a model for studying the requirements needed for immune mediated destruction of tumor cells and gives us the opportunity to dissect the important aspects of T-cell, tumor and host biology and their contributions to effective anti-tumor responses. Furthermore, TMEV is easy to grow and is not known to be a human pathogen despite being used extensively in the scientific community. This makes TMEV an interesting candidate for further development as an effective, safe and inexpensive vaccination for immunotherapy.

## Materials and Methods

### Ethics Statement

This study was carried out in accordance with the recommendations in the Guide for the Care and Use of Laboratory Animals of the National Institutes of Health and according to the Institutional Animal Care and Use Committee of Mayo Clinic. These studies were specifically approved by IACUC under protocols A38409 and A43310.

### Mice

 C57BL/6, C57BL/6-Tg(Ins2-OVA)59Wehi/WehiJ (RIP-OVA ) and C57BL/6-Tg(TcraTcrb)1100Mjb/J (OT-1) mice were obtained from Jackson Laboratory (Bar Harbor, ME) and maintained in the institutional animal facility. H-2D^bm14^ (bm14) [12] were bred and maintained in the same facility. All animals were housed and cared for according to institutional and NIH guidelines for animal care and use. Animals were euthanized when tumor sizes exceeded 225 mm^2^.

### Cell lines

B16, L929 and BHK cells were maintained in DMEM (GIBCO Invitrogen, Grand Island, NY) and EL4 were maintained in RPMI (GIBCO/Invitrogen) containing 10% Cosmic™ calf serum (Hyclone, Logan, UT). B16-OVA lines were maintained in the same media supplemented with 10 mg/mL Geneticin (GIBCO Invitrogen, Grand Island, NY).

### Reagents

PerCP labeled anti-mouse CD45 and FITC labeled anti-mouse CD8 were purchased from BD Biosciences (San Diego, CA, USA). PE labeled H-2K^b^/OVA_257_ tetramers (part no. T03000) were purchased from Beckman Coulter (Brea, CA, USA). PE labeled H-2D^b^/VP2_121_ tetramers were kindly provided by the NIH Tetramer Core at Emory University (Atlanta, GA, USA). VP2_121_ (FHAGSLLVFM), OVA_257_ (SIINFEKL) and E7_49_ (RAHYNIVTF) peptides were synthesized by Elim Biopharm (Hayward, CA, USA).

### Vaccine generation and quantification

Epitope modified vaccine was generated from a cDNA clone of the Daniel's strain of TMEV, pDAFL_3_ [13]. Two epitopes including the H-2D^b^ epitope gp_33_ from LCMV and the H-2K^b^ epitope OVA_257_ were introduced into an Xho I restriction site within the leader sequence of TMEV. The two epitope fragments were amplified independently and then linked through PCR splicing by overlap extension [14], digested with Xho I and ligated into the leader sequence at position 1221 of the TMEV genome. As described previously [13], T7 RNA transcripts were generated and transfected into BHK cells using electroporation. Five days later supernatant and cells were harvested and assayed for the presence of virus using plaque assays on L929 cells. Once viral titers were determined, high titer virus for injection was generated by inoculating flasks of BHK at an MOI of 0.01 [15]. Final virus titers were determined by plaque assay. Mice were inoculated with either 5×10^5^ PFU intracranially or with 5×10^6^ PFU intraperitoneally.

Brains and spinal cord from mice infected with TMEV-wt or TMEV-L/OVA for 6 days were homogenized, sonicated and clarified. Homogenates were tested for viral titer using plaque assays described previously [16].

The 7900HT Fast Real-Time PCR System (Applied Biosystems, Carlsbad, CA, USA) was used to quantify viral RNA in 24 day infected brain and spinal cord homogenates from mice inoculated with TMEV-wt or TMEV-L/OVA. RNA was isolated using TRIzol Reagent (Invitrogen, Carlsbad, CA, USA) and reverse transcribed using the Superscript cDNA synthesis kit (Invitrogen). Reaction was set up using the Fast SyBR Green Master Mix Kit (Applied Biosystems). cDNA was amplified using primers specific for mouse actin (F – 5′CTGGCACCACACCTTCTACAATGAGCTG and R– 5′GCACAGCTTCTCTTTGATGTCACGCACGATTTC) and for viral protein 2 (VP2) of TMEV (F-5′TGGTCGACTCTGTGGTTACG and R-5′ GCCGGTCTTGCAAAGATAGT). Cycling conditions were as follows: 50°C for 2 minutes, 95°C for 10 minutes followed by 40 cycles of 95°C at 15 seconds then 55°C for 1 minute. Amplification curves and crossing point thresholds were based on SYBR Green incorporation. Samples were normalized to actin and data are reported as fold increase over background.

### In vivo and in vitro killing assay

Chromium release assays were used to determine cytotoxic cell killing from brain infiltrating and tumor infiltrating lymphocytes. For mice intracranially infected with virus, brains were recovered from 6 day TMEV infected mice and brain infiltrating lymphocytes (BIL) were isolated via percoll gradient as described previously [5]. Tumor infiltrating lymphocytes (TIL) were recovered by removing implanted tumors and physically disrupting the tumor before passing through a 45 µm cell strainer. To enrich for lymphocytes, disrupted tumor cells were allowed to settle for 15 minutes before harvesting lymphocytes. Peptide pulsed and unpulsed EL4 cells were used as targets in a standard 4 hour chromium release assay.

In vivo killing assays were used to assess target cell specific killing after induction of cytotoxic T-cells [17]. Briefly, we labeled 3 target populations of splenocytes with high concentration carboxyfluorescein succinimidyl ester (CFSE), low concentration CFSE and with a third label PKH26 (Sigma-Aldrich, St. Louis, MO, USA). These three populations were labeled with OVA_257_, E7_49_ or VP2_121_ before intravenous injection into TMEV vaccinated mice. Splenocytes were harvested and individually analyzed by flow cytometry to determine the percent killing of individual populations of labeled cells. Percent killing was normalized to the irrelevant peptide control population pulsed with E7_49_ peptide.

### RIP-OVA model of diabetes mellitus

RIP-OVA mice received 5×10^6^ PFU of TMEV-wt or TMEV-L/OVA vaccine on the same day as adoptive transfer of OT-1 CD8+ cells or without transfer. Mice receiving intravenous transfer were given 1×10^7^ purified CD8+ T-cells enriched with the mouse CD8a+ T-cell isolation kit (Miltenyi Biotec, Bergisch Gladbach, Germany). Glucose levels were monitored daily using a Lifetouch glucomoter (Lifescan, Milpitas, CA U.S.A). After two successive readings of >500 mg/dL, mice were sacrificed for histologic analysis and insulin immunohistochemistry. Pancreata were harvested and fixed in formalin before paraffin embedding and sectioning. Sections were stained with hemotoxylin and eosin using standard procedures. Microscopic analysis and imaging was performed on an Olympus DP70 camera attached to an Olympus AX70 research microscope (Olympus America Inc., Center Valley, PA).

### Tumor growth, recovery and analysis

B6 mice were challenged in the right flank with 5×10^5^ B16 or B16-OVA tumor cells and were treated with viral vaccines on the day of tumor challenge or on day 9 after challenge. Tumors were measured in two dimensions every other day. Tumor index is reported as the square root of the product of both dimensions using millimeters. For tumor recovery experiments, tumors were recovered and disrupted with the plunger from a 3 mL syringe, then passed through a nylon mesh filter. An aliquot of cells was used to isolate RNA using the RNEasy kit (Qiagen, Germantown, MD) to determine expression of the transfected ovalbumin gene by qRT-PCR. Cells were plated in 12 well plates at 2×10^5^ cells per well in media supplemented with or without G418 (Invitrogen). Three days later, cells were washed, fixed and stained with crystal violet. Plates were scanned with a flat bead scanner and percent of area covered by crystal violet stained cells was determined using ImageJ (National Institutes of Health; http://rsbweb.nih.gov/ij/index.html).

### Statistics

Statistical analysis was performed using SigmaStat3.1 software (Systat Software, San Jose, CA). Non-parametric ordinal data were analyzed by rank-sum test and categorical data by Fisher Exact test. Normally distributed data were analyzed by student's t-test or by one-way or two-way ANOVA with pairwise comparisons done by Student-Neuman-Keuls Method. Significance was determined by P values less than 0.05.

## Results

### Epitope modified TMEV replicates in vitro but does not manifest a persistent infection

**Figure 1:**
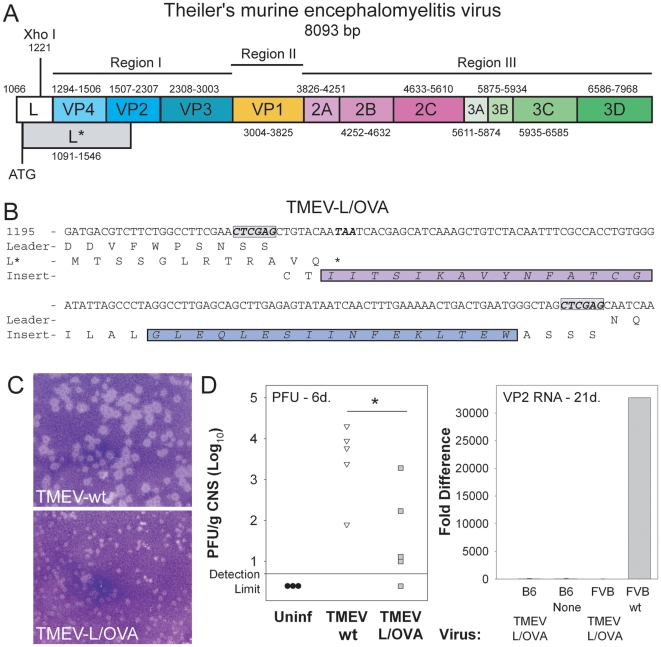
The generation of epitope modified TMEV vaccine. (A) The genome of TMEV contains an Xho I restriction site within the leader sequence which can be used for insertion of MHC class I peptide epitopes. (B) Sequence of LCMV and ovalbumin linked epitopes inserted into the Xho I restriction site of the pDAFL_3_ vector. (C) Productive infection and plaques from wild-type and modified TMEV virus. (D) Plaque assay (left) of virus recovered from the brains of mice infected with virus for 6 days (*p = 0.014). Absence of detectable viral transcripts in the brain 21 days after inoculation with TMEV-L/OVA vaccine in B6 and FVB mice (right).

We sought to use TMEV as a vector for driving an immune response against introduced epitopes. Based on previous observations that introduction of epitopes within a Xho I restriction site (Figure 1A) in the leader sequence could be used as a vector for foreign gene delivery, we engineered the pDAFL3 clone [13] to encode two model epitopes in the leader sequence of the TMEV clone. Two model epitopes, the H-2D^b^ epitope gp_33_ from LCMV and the H-2K^b^ epitope OVA_257_ from ovalbumin (Figure 1B) were introduced into the TMEV genome, positive stranded RNA was generated and transfected into BHK cells to generate epitope modified TMEV-L/OVA virions.

To verify that the modified virus was viable and infectious we performed plaque assays on transfected BHK supernatants to determine viral titers. The TMEV-L/OVA was raised to a titer 4×10^5^/ml supernatant as compared to 2×10^7^ for wild-type virus, suggesting a possible loss in virulence. The size of individual plaques generated by the TMEV-L/OVA virus was notably reduced compared to a cultured passage of wild-type TMEV-DAV (Figure 1C). Since intracranial infection is often used to determine virulence of TMEV, we injected viruses into the brains of C57BL/6 mice and determined the titer of TMEV-wt and TMEV-L/OVA by plaque assay from brain and spinal cord homogenates after six days of infection. The viral titers TMEV-L/OVA maintained in the CNS after 6 days of infection were significantly reduced compared to TMEV-wt (Figure 1D). The Daniel's strain of TMEV can persist in the CNS of genetically susceptible hosts [2]. To determine whether our modified virus would persist, we infected resistant C57BL/6 or susceptible FVB mice for 21 days. As expected, infection of the resistant strain with TMEV-L/OVA did not lead to persistence as demonstrated by the lack of viral specific transcripts. Further, the modified virus did not persist in the susceptible FVB strain either (Figure 1D). In comparison, FVB infected with TMEV-wt maintained TMEV transcripts at a level that was 30,000 fold higher than background. This demonstrates that while TMEV-L/OVA is still infectious, its inability to persist in susceptible hosts and its reduced virulence show that the virus is substantially attenuated.

### Generation of CD8+ effectors against epitopes introduced into the TMEV genome

**Figure 2:**
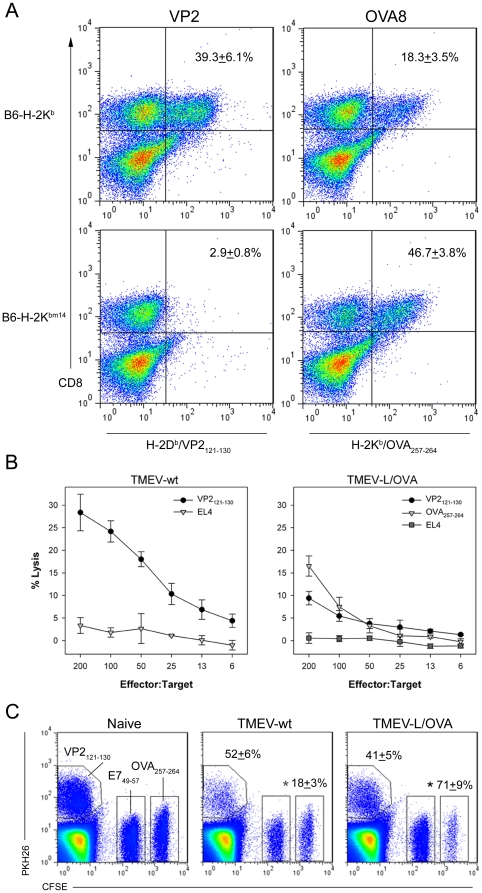
Generation of epitope specific CD8+ T-cell responses with TMEV-L/OVA. (A) FACS analysis of brain infiltrating lymphocytes (BIL) from mice infected with TMEV-L/OVA for 6 days. The proportion of OVA_257_ specific T-cells increases in the absence of viral specific CD8+ T-cells (p<0.001). (B) In vitro cytotoxic activity of BIL as measured by a 4 hour chromium release assay using VP2_121_ and OVA_257_ peptide pulsed targets. (C) OVA_257_ specific in vivo killing of labeled target cells in 6 day TMEV-L/OVA infected mice was increased compared to TMEV-wt (p = <0.001).

Since we have shown that the epitope modified TMEV is still competent in its ability to infect cells, we next determined whether this virus could generate CD8+ effector cells against the introduced epitopes. Intracranial injection of TMEV-wt into C57BL/6 mice leads to an immunodominant CD8+ effector cell population that is specific for the H-2D^b^ specific viral epitope VP2_121_ [5]. Although not intended as a route of therapy, intracranial (ic) infection provides a convenient way to monitor the induction of tissue infiltrating inflammatory cells. Our engineered virus contains epitopes for the H-2D^b^ restricted epitope gp_33_ and the H-2K^b^ epitope OVA_257_. We infected mice ic with TMEV-L/OVA and evaluated the CNS infiltrating CD8+ T-cell population by using tetramers specific for gp33, OVA_257_ and VP2_121_. CD8+ cells staining with K^b^:OVA_257_ tetramer, as well as cells staining with the VP2_121_:D^b^ tetramer were detected readily (Figure 2A).

The coincidence of two immunodominant responses against peptide epitopes encoded in the virus genome raised the question of whether competition among epitopes included in the vaccine could alter immunization efficiency. In a previous study, we demonstrated that the H-2D^b^ mutant bm14 is unable to mount a response to the VP2_121_ epitope due to changes in antigen presentation by the bm14 molecule [18]. We used this model system to determine whether responses to the H-2K^b^ allele would be enhanced in the absence of a response to the D^b^ restricted virus-encoded VP2_121_ epitope. Similar to previous findings with TMEV-wt (26), there was no CD8+ T-cell response to VP2_121_ by the bm14 mice challenged with TMEV-L/OVA. However, the response to the ovalbumin epitope represented a greater proportion of the CD8+ T-cells present in the brains of bm14 mice infected with TMEV-L/OVA for 6 days (48% of the CD8 T cells) compared to infection of wild-type mice (18% of the CD8 T cells). Assuming that the CD8+ T cell responses to epitopes encoded by TMEV-L/OVA virus by B6 wildtype mice are independent (i.e. 39.3% anti-VP2, 18.3% anti-OVA, and 42.4% undefined), deletion of the VP2 specific response would have been expected to result in 30% of the remaining CD8+ T cells having OVA specificity (18.35/[18.35+42.4%]), not the observed 48%. Therefore, it seems likely that the presence of more than one immunodominant epitope in a virus might result in competition, influencing the effectiveness of the vaccine against individual epitopes of interest. This important point will need further investigation, as immunization against multiple epitopes is a frequent goal of designs of T cell vaccines.

Having demonstrated that CD8+ cells can be elicited specific for an introduced peptide epitope with our modified virus, we next tested whether this response was effective at generating cytotoxic effectors. To address this, we harvested lymphocytes from the CNS of mice intracranially infected with TMEV-wt or with TMEV-L/OVA and tested their ability to lyse chromium labeled target cells. As shown previously, lymphocytes harvested directly from TMEV-wt infected CNS kill VP2_121_ peptide pulsed targets very effectively (Figure 2B). Similarly, the epitope modified virus was able to elicit cytotoxic lymphocytes that could target directly both the dominant viral epitope and the introduced ovalbumin epitope, demonstrating the virus' effectiveness at driving cytotoxic effector cell responses to introduced antigens.

Since we were able to demonstrate effective killing by CNS infiltrating lymphocytes directly ex vivo, we wanted to determine whether an intraperitoneal injection of TMEV-L/OVA could generate effective killing in vivo. We injected TMEV-wt and TMEV-L/OVA viruses directly into the peritoneum and on day six challenged the mice with peptide pulsed splenocytes to determine the effectiveness of this vaccine against in vivo labeled targets. Consistent with the chromium release assay, TMEV-wt vaccinated mice generated robust responses against VP2_121_, whereas the TMEV-L/OVA vaccinated mice killed both VP2_121_ and OVA_257_ pulsed targets. These data indicate that the epitope modified vaccine can elicit effector cells against introduced antigens and that these responses can effectively clear target cells presenting the targeted antigens in vivo. Administration of the virus via intracranial, intraperitoneal or intravenous route yielded comparable results (data not shown).

### Targeted tissue damage induced in vivo with modified TMEV vaccine

**Figure 3:**
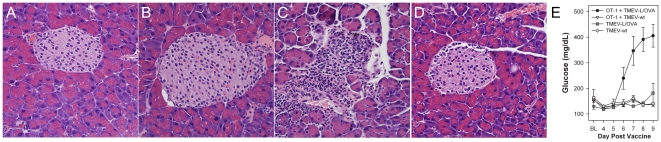
Induction of diabetes with TMEV-L/OVA using RIP-OVA mice given OT-1 T-cell transfer. Representative pancreatic islets from RIP-OVA mice given TMEV-wt (A) or TMEV-L/OVA (B) without OT-1 transfer. (C) Pancreatic islet infiltration observed in OT-1 transferred RIP-OVA receiving TMEV-L/OVA vaccine compared to TMEV-wt vaccine (D). (E) Blood glucose levels observed in RIP-OVA mice given TMEV-wt or TMEV-L/OVA vaccines. Increased blood glucose was observed on day 6 (p = 0.007), 7 (p<0.001), 8 (p<0.001) and 9 (p<0.001) in mice receiving both OT-1 transfer and TMEV-L/OVA vaccine compared to transfer with TMEV-wt.

Having established that epitope modified TMEV can elicit CD8+ T-cell specific immunity, we asked whether systemic vaccination could activate cytotoxic effectors that could infiltrate and destroy antigen bearing cells in solid tissues. We used adoptive transfer of OT-1 T-cells into RIP-OVA mice as a model, visualizing cellular immune attack against beta cells in the pancreas. In the absence of OT-1 transferred T-cells, RIP-OVA mice injected with either TMEV-wt or with TMEV-L/OVA failed to develop pancreatic pathology or lymphocyte infiltration into pancreatic islets (Figure 3A and B). TMEV-L/OVA vaccination of RIP-OVA mice reconstituted with OT-1 T-cells generated robust pancreatic islet inflammation (Figure 3C), whereas TMEV-wt vaccination had no effect (Figure 3D). The ability of the infiltrating T cells to damage the islets was evident as the mice receiving both OT-1 transfer and TMEV-L/OVA vaccination developed high glucose levels, characteristic of type 1 diabetes. We conclude from this study that TMEV-L/OVA vaccination activates CD8+ T-cells which can specifically target and damage antigen expressing tissues.

### Epitope modified TMEV vaccine edits ovalbumin expressing tumors and inhibits B16-OVA outgrowth

**Figure 4:**
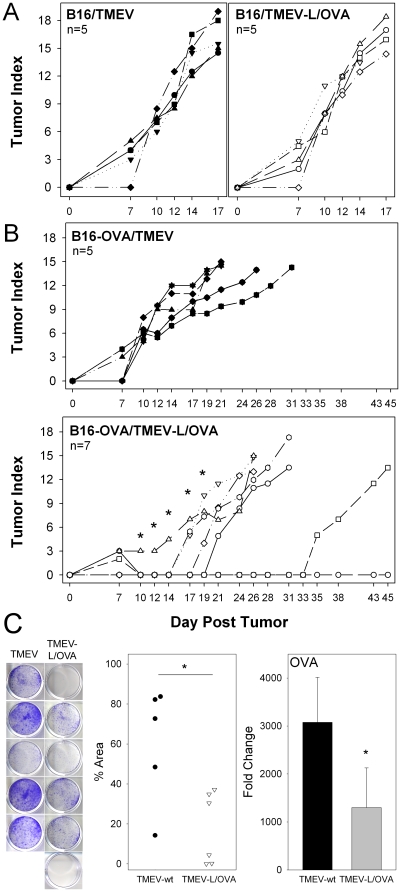
MHC class I epitope specific protection and targeting of B16-OVA melanoma. (A) Vaccination with TMEV-wt or TMEV-L/OVA virus did not delay tumor outgrowth using the parental B16 tumor model. (B), Tumor outgrowth in mice challenged with B16-OVA tumor and vaccinated with TMEV-wt (top) or with TMEV-L/OVA (bottom). Tumor sizes were significantly different in these treatment groups on days 10 through 19 (* designates p<0.05). (C) G418 resistance and growth of tumor cells recovered from mice vaccinated with TMEV-wt or with TMEV-L/OVA (left). Quantitation of cresyl violet stained tumor cells recovered from vaccinated mice. Data expressed as the percent of well area containing stained tumor cells from TMEV-wt and TMEV-L/OVA vaccine treated mice (middle) (p<0.05). RNA isolated from recovered tumors was analyzed by qRT-PCR for the presence of ovalbumin specific transcripts (p = 0.009).

Since we were able to effectively activate OT-1 cells in vivo using the TMEV-L/OVA virus, we asked whether we could activate a polyclonal T-cell response in the absence of non-transgenic T-cells using the native repertoire in B6 mice. We used the melanoma tumor model B16-OVA to determine whether the epitope modified vaccine could effectively target the tumor and inhibit outgrowth. Since oncolytic viral therapy is often used to directly target and kill tumor cells, we first asked whether TMEV or our modified virus could directly target and kill the B16 melanoma line, which does not express the ovalbumin epitope, OVA_257_, or the viral epitope VP2_121_. We challenged B6 mice with the B16 tumor line in the flank of the hind leg and then vaccinated them ip with either the TMEV-wt or TMEV-L/OVA viruses. Neither virus was able to inhibit tumor outgrowth (Figure 4A), which proceeded comparably to tumor growth we have observed repeatedly in mice not receiving virus challenge (data not shown).

We then asked whether these viruses could be used to elicit tumor specific immunity against the B16-OVA tumor line. Mice were challenged with B16-OVA on the same day as vaccination with the wild-type and epitope modified virus. By day 14 all of the TMEV-wt treated mice had measurable tumors, whereas only one mouse in the TMEV-L/OVA treated group had a measurable tumor (Figure 4B). The durability of the tumor inhibition varied substantially among the mice treated with the TMEV-L/OVA virus, raising the possibility that a stochastic event was contributing to resistance to the tumor or tumor escape. To determine whether tumors were avoiding sterilization by escaping immune selection, we analyzed tumors that grew out of TMEV-wt and TMEV-L/OVA immunized mice. The B16-OVA tumor was prepared originally by introduction of transgenes expressing chicken ovalbumin and a *neomycin resistance gene* from *E. coli* transposon Tn5. By growing single cell suspensions of harvested tumors and assessing them for resistance to G418, the presence of the antibiotic resistance gene co-expressed on the ovalbumin construct used to generate the B16-OVA tumor cell line was assessed. We found that mice treated with the TMEV-wt virus primarily grew tumors that were resistant to G418, consistent with the presence of the ovalbumin vector. However, the tumors from TMEV-L/OVA vaccinated animals had a dramatic reduction in their resistance to G418 (Figure 4C). Finally, semi-quantitative RT-PCR analysis of tumor cells demonstrated that there was a significant reduction in expression of ovalbumin specific transcripts in the TMEV-L/OVA treated mice. These data indicate that the virus vaccine induces effective immunity primarily targeting ovalbumin expressing cells and that immune editing has allowed tumors which have lost expression of the transgene containing both the OVA antigen and neomycin resistance to predominate. This form of immune editing of the B16-OVA tumor was demonstrated initially using OVA specific OT-1 T cells by our colleagues, Karen Kaluza and Richard Vile, Mayo Clinic (personal communication and manuscript in preparation).

### Treatment of 9 day established B16-OVA tumors with epitope modified TMEV leads to robust tumor specific immunity, enhanced antigen specific tumor infiltrating lymphocytes and delays tumor outgrowth

**Figure 5:**
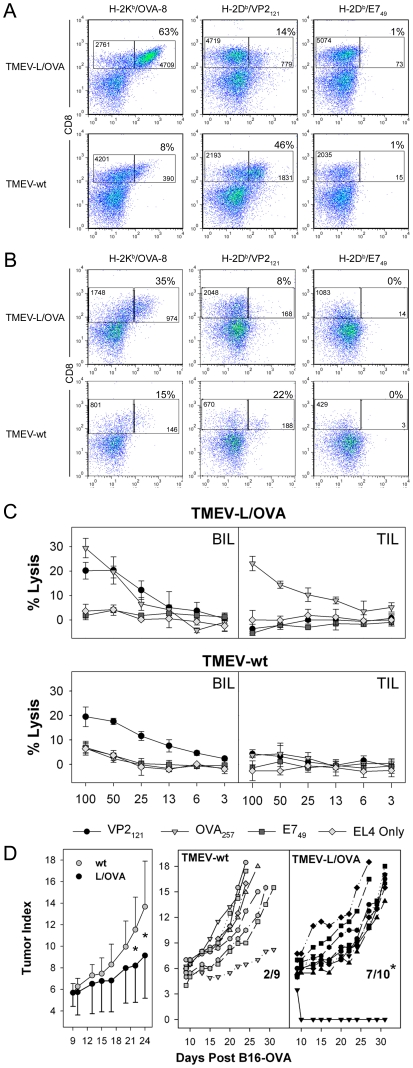
Delayed tumor outgrowth using TMEV-L/OVA vaccine in a therapeutic model of established tumor burden. FACS analysis of 6 day BIL (A) and TIL (B) from mice given TMEV vaccines on day 9 after B16-OVA implantation. Lymphocytes were assessed for the presence of OVA_257_, VP2_121_ and E7_49_ specific CD8 T-cell responses. Percentages are the percent of tetramer specific CD8 cells. Numbers represent the absolute numbers of cells per 100,000 events. (C) Four hour chromium release assay using day 15 TIL from mice treated with TMEV-L/OVA or TMEV-wt 9 days after B16-OVA challenge. (D) (left) Observed tumor growth in mice treated with TMEV-wt or TMEV-L/OVA. (p<0.05 at day 21 and 24). Tumor outgrowth and survival of individual animals treated with TMEV vaccines. Significant differences in survival were observed between the treatment groups at the conclusion of the 30 day observation period (p<0.05).

Because we found that TMEV-L/OVA vaccination has an effect on B16-OVA outgrowth when tumor was given at the time of virus challenge, we wanted to determine whether this treatment could be effective in an established tumor model. We challenged mice with B16-OVA in the flank of the hind leg nine days prior to infection with TMEV-wt or TMEV-L/OVA virus and monitored the immune response to the OVA_257_ and VP2_121_ epitopes. Although systemic infection with the TMEV vaccine induced robust CTL, in these experiments we introduced this virus intracranially to facilitate monitoring the recruitment and migration of viral specific and ovalbumin specific CD8+ T-cells into the site of infection. Tetramer analysis demonstrated a robust OVA_257_ specific infiltration in the brain of infected mice, which was dramatically reduced in the TMEV-wt mice. Both viruses elicited VP2_121_ specific CD8 cells compared to irrelevant E7 tetramer, however the TMEV-L/OVA response to the VP2_121_ epitope was reduced (Figure 5A), indicating that the TMEV-L/OVA response focused on the ovalbumin epitope in mice bearing nine day tumors expressing this antigen.

Since this vaccine generated strong CD8 immunity at the site of infection, we wanted to determine whether vaccination with TMEV-L/OVA also altered the quality of the tumor infiltrating lymphocyte response in established B16-OVA melanomas. Thirty-five percent of the CD8+ tumor infiltrating T cells stained brightly with OVA_257_:K^b^ tetramer B16-OVA tumor, indicating that the epitope-modified vaccine delivered at a remote site can influence the quality of the T-cell response at the tumor. VP2_121_- specific T-cells were found in the tumor and none of the tumor infiltrating T cells stained with the irrelevant E7 tetramer (Figure 5B), demonstrating the specificity of T-cell infiltration and retention in the tumor.

One hallmark of effective tumor therapy is the development of cytotoxic lymphocyte responses specific to the tumor. We challenged mice bearing 9 day tumors with TMEV-wt or TMEV-L/OVA ip and assessed the tumor infiltrating lymphocyte (TIL) population for its ability to kill OVA_257_ peptide pulsed targets 6 days after intraperitoneal challenge with either virus. We found that TIL harvested from the TMEV-L/OVA treated mice generated effector cells that could effectively target and kill OVA_257_ peptide pulsed targets, whereas TMEV-wt treatment failed to induce this response (Figure 5C).

Because we have established that tumor specific cytotoxic cells were infiltrating the tumor, we wanted to assess whether this could influence tumor outgrowth. We monitored tumor outgrowth in mice seeded with B16-OVA 9 days prior to treatment with either virus. Twelve and 14 days after a single inoculation of TMEV-L/OVA virus ip, we observed a significant reduction in tumor size (65% reduction) compared to the tumors in mice treated with the wild type virus (Figure 5D). Further, this delay in outgrowth significantly increased the survival of the TMEV-L/OVA group at 30 days post tumor challenge (Figure 5D), where 7 of 10 animals had not developed tumors exceeding 225 mm^2^ (length, width) compared to only 2 of 9 mice in the TMEV-wt treated group. This reduction in tumor burden and delay in outgrowth indicates that the epitope-modified vaccine can be used to induce immunotherapeutic responses against established tumors, raising the possibility of relevance for further development as a candidate vaccine for treatment of human disease.

## Discussion

Successful active immunotherapy strategies will have to overcome a number of factors limiting effective immune responses in cancer patients, including immune suppressive tumor micro-environments, limitations in the immune repertoire resulting from mechanisms of tolerance, and the need to activate strong immune responses in vivo. Here we address one of these, the requirement to activate robust cellular immunity against cancer associated antigens. We pursued the hypothesis that directing potent anti-viral immunity against tumors results in sterilizing immunity as we evaluated the picornavirus, TMEV as a candidate for cancer vaccine development.

Several characteristics of TMEV make this virus an attractive candidate as a vaccine vector for immunotherapy. One property that makes it particularly attractive is that it is not a natural human pathogen [19]. Although cardioviruses were thought to be exclusively infective in rodents, a recently identified virus species in humans shows that asymptomatic infections with the cardiovirus member Saffold virus are highly prevalent in humans [20]. Furthermore, the precise receptor for TMEV has not been determined [21], and this virus can infect human cells in vitro indicating that binding to a conserved receptor could cause a subclinical infection in humans. This leaves open the possibility that strong T-cell responses to introduced antigens could be induced with a TMEV vaccine.

Another attractive property is the relatively small genome that can be easily manipulated. One of the drawbacks to the use of larger viral vectors for vaccine development [22] is that the human immune system may focus on several viral epitopes rather than introduced epitopes. TMEV is composed of only 12 unique protein antigens and infection of resistant C57BL/6 mice leads to T-cell responses that result in up to 70% of activated CD8+ T-cells focusing on one major epitope [5]. Simple modifications of this epitope [23] may subvert CD8 responses against the virus allowing the immune system to focus on the introduced targets of interest. This has important implications as we find that responses against immunodominant epitopes encoded in the vaccine can compete with each other. Future studies aimed at eliminating potential viral epitopes recognized by human MHC restricted T-cells could tailor the virus for effective targeting of antigens of interest. Further, the predominant antibody neutralization sites for TMEV have been identified [24], providing approaches for controlling virus infectivity. Because blocking antibodies are highly effective in preventing TMEV infection, passive transfer of antibody could help control virus infection and elimination of antibody epitopes could enhance ability to revaccinate and give rise to increased cellular immunity.

Our studies demonstrate that the introduction of a single epitope can have a significant effect on tumor outgrowth and survival; however tumors in most mice did continue to grow and demonstrated immune editing as shown by a decrease in ovalbumen expression in vaccine treated animals. It is known that tumors can use several mechanisms to avoid the immune response including down-regulation of target antigens [25]. Therefore, the most effective vaccines will be those which can incorporate multiple antigens including those that are critical to tumor survival. Prior studies have shown that the 239 amino acid protein enhanced green fluorescent protein can be introduced into the genome of TMEV and that infectious virions can be generated that express this protein [6]. This demonstrates the potential for this vaccine to be enhanced by driving T-cell responses to multiple peptide antigens, increasing its effectiveness as a vaccine.

One concern with modifying viruses for use as vectors is the possibility that the modified virus may have increased virulence in the host. Our study demonstrates that modifications we have introduced have the opposite affect. Several virulence factors in the capsid and non-capsid regions of the TMEV genome have been previously identified [26], [27], [28], including the leader peptide [29]. We have introduced the H-2K^b^ restricted epitope OVA_257_ into the leader sequence of TMEV at position 53 of this 76 amino acid protein. This non-capsid viral protein has been shown to be important for inhibiting type I interferon responses that are important for innate immune responses to the virus as well as for viral assembly in L cells [30], [31]. Since productive virus can be generated in BHK cells and viruses generate plaques on L cells, it is unlikely that this insertion affects the function of the leader sequence in viral assembly. However, this insertion may interfere with its role in inhibition of type I interferon induction potentially through its interaction with IRF3 [32] which may explain the attenuated phenotype observed with the TMEV-L/OVA virus. Future experiments that address the function of the epitope modified leader sequence will clarify the role of this disruption in inhibiting the type I interferon response.

Another concern with using modified virus vaccines is the potential for recombination between species within the picornavirus family which could potentially lead to a more virulent species [33]. The rodent viruses TMEV and encephalomyocarditis virus were the only known members of the cardiovirus genus until the discovery of Saffold virus in 2007 [34], leaving open the possibility that novel TMEV like viruses may serve as potential genetic donors for modified TMEV vaccines. However, recombination within the picornavirus family appears to be limited to viruses within a species and intra-species recombination is very rare [35]. Most picornavirus infections are asymptomatic however if isolated only 5% of viral isolates can be expected to contain two or more strains [33], making the possibility of recombination between an unknown cardiovirus very unlikely and strengthening the practical use of TMEV as a vaccine vector.

One important aspect that is implicit in vaccine design is that the immune response elicited is mechanistically appropriate to the intended target. Previous work with TMEV has demonstrated that it is a very strong activator of cytotoxic T-cell responses [36] and cytotoxicity is dependent on perforin contained in CD8+ T-cells [37]. The mechanism of killing induced with perforin and granzymes leads to apoptosis and clearance of targeted cells, a mechanism favored by investigators designing immune therapies for the treatment of cancer. The current work demonstrates that cytotoxic T-cells can be induced to T-cell epitopes that are artificially introduced into the viral genome. Although infection with TMEV-L/OVA is a potent inducer of virus specific cytotoxic T-cell responses at the site of infection, tumor specific T-cells also invade the tumor at distant sites further strengthening its potential use as a vaccine vector.

Our findings show that TMEV-L/OVA treatment inhibits tumor growth and also increases the proportion of tumor specific lymphocytes in the tumor and in the brain after intracranial injection of tumor bearing mice. Although we did not make discreet measurements of the absolute number of OVA_257_ specific CD8+ T-cells in the brain after TMEV-L/OVA treatment, our data show that there was an increase in the quantity and density of antigen specific T-cells inside the tumor, a more promising indicator of a positive therapeutic effect [38]. Our methods for isolating lymphocytes included an enrichment step for brain infiltrating lymphocytes only. We analyzed a fixed number of events from the lymphocyte enriched brain homogenate and found that a higher proportion of T-cells were OVA_257_ specific after TMEV-L/OVA treatment, however since the competing VP2_121_ response in the wild-type DA and L-OVA infected mice were not normalized by using a given volume, it is difficult to determine whether absolute numbers CD8+ brain infiltrating T-cells were different. However, the tumor infiltrating lymphocyte prep was derived from a whole tumor cell suspension where between 95% and 97% of the cells were negative for the common lymphocyte antigen CD45, presumably tumor cells, thus providing a normalization factor. Additionally, tumor sizes were not significantly larger or smaller 6 days after vaccine treatment, demonstrating that the absolute number and density of T-cells in the tumor were greater in the TMEV-L/OVA group.

The presence of tumor invading T-cells in cancer is not an uncommon occurrence; however mechanisms employed by the tumor itself help to inhibit further tumor invasion as well as the specific effector functions of T-cells. Vaccination with TMEV-L/OVA induces the enhanced infiltration of T-cells into target tissues and this infiltration was associated with dramatic induction of diabetes using the RIP-OVA model and also led to an increase in cytotoxicity of tumor infiltrating lymphocytes with an accompanied decline in tumor outgrowth and increased survival. Although these were not overt cures of established tumors, the ability to target specific tumor antigens was apparent as the outgrowth of tumors in vaccinated mice showed substantial reductions in overall tumor antigen expression, as well as a decrease in the number of tumor cells that expressed antibiotic resistance associated with the ovalbumin transgene introduced into the B16 tumor line. Future experiments designed to optimize the tumor specific immune response through modification of the viral genome may increase this virus' efficacy in established tumor models.

Several viruses in the picornaviridae family have been studied for their ability to selectively target, infect and lyse tumor cells [39], [40], [41]. However, TMEV-L/OVA vaccines indirectly target tumor cells by eliciting cytotoxic T-cell immunity that kills tumor cells with exquisite specificity. Although wild-type TMEV can infect B16 in vitro (data not shown), no effect on tumor growth was observed when either wild-type or TMEV-L/OVA virus vaccines were given to B16 bearing mice. Further, vaccination of B16-OVA bearing mice with wild-type virus did not alter tumor growth kinetics, suggesting that TMEV does not promote epitope spreading to tumor associated antigens. Only the epitope modified virus, which can specifically induce cytotoxic T-cells that target tumor associated antigens, had an effect on the outgrowth of tumors.

Optimal vaccines for immunotherapy must be easily amenable to modification, must target multiple antigens, and most importantly, must elicit the type of immunity required to eradicate infectious agents or to eliminate cancer cells. TMEV represents an attractive new candidate that can be easily manipulated with standard techniques, yet elicits strong T-cell immunity that can be harnessed and targeted towards tumor cells. These characteristics make TMEV an attractive vaccine vector for the induction of cellular immunity against tumors and may provide a novel vaccine strategy for targeting tumors that have not responded to existing chemotherapy and vaccination strategies.
